# Focal Monomorphic Ventricular Tachycardia As The First Manifestation Of Amyloid Cardiomyopathy

**Published:** 2010-03-05

**Authors:** Srikanth Seethala, Sandeep Jain, N. Paul Ohori, Sara Monaco, Joan Lacomis, Frederick Crock, Jan Nemec

**Affiliations:** 1 Department of Internal Medicine, University of Pittsburgh, 200 Lothrop St, Pittsburgh PA 15213; 2 Cardiovascular Institute, University of Pittsburgh, 200 Lothrop St, Pittsburgh PA 15213; 3 Department of Pathology, University of Pittsburgh, 200 Lothrop St, Pittsburgh PA 15213; 4 Department of Radiology, University of Pittsburgh, 200 Lothrop St, Pittsburgh PA 15213

**Keywords:** amyloid cardiomyopathy, ventricular tachycardia, AICD

## Abstract

52-year-old patient presented with palpitation and well tolerated monomorphic ventricular tachycardia. He had normal echocardiogram and coronary angiogram 3 months prior to presentation. Surface EKG revealed regular wide-complex tachycardia with right bundle branch block morphology and right inferior axis. In conjunction with recent negative cardiac evaluation, this suggested idiopathic focal ventricular tachycardia from anterolateral basal left ventricle. CARTO based activation mapping confirmed the presence of VT focus in that area. Radiofrequency ablation at the site of perfect pacemap resulted in a partial suppression of the focus. Echocardiogram was subsequently performed because of progressive dyspnea. It revealed asymmetrical thickening of posterolateral left ventricle, with delayed enhancement on contrast magnetic resonance imaging. Fine needle aspiration of abdominal fat stained with Congo red confirmed the diagnosis of systemic AL amyloidosis due to IgG λ-light chain deposition. Consequently, the patient underwent placement of implantable defibrillator and hematopoetic stem cell transplantation. He remains in excellent functional status 18 months after presentation.

## Case Report

**Figure 1:**
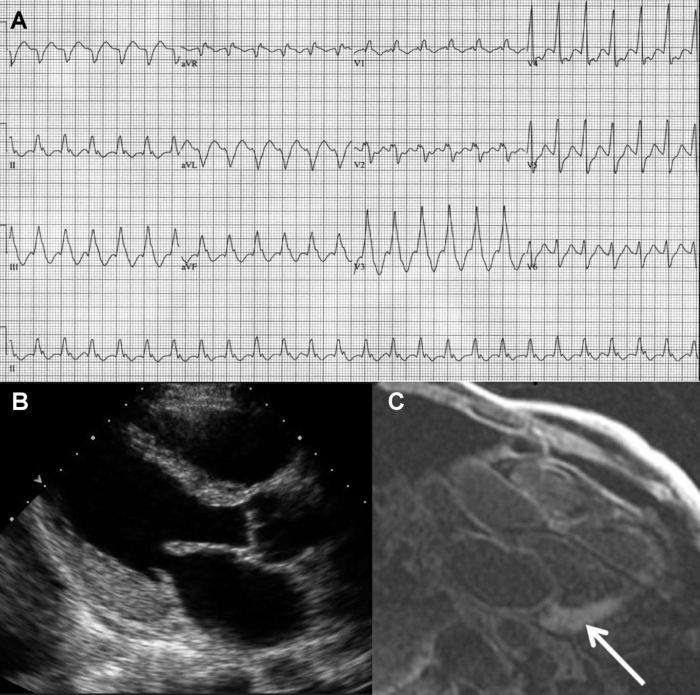
A Standard electrocardiogram of the presenting arrhythmia. No definite atrial activity can be seen and the QRS morphology is suggestive of ventricular tachycardia with a focus in basal anterolateral LV. This was confirmed during radiofrequency ablation. B Asymmetrical thickening of LV wall is seen on echocardiogram in parasternal long axis view (diastolic frame). The basal posterolateral wall thickness is 15 mm, while the thickness of the intervetricular septum appears normal. C Transmural delayed enhancement of posterolateral wall of the LV (arrow) on magnetic resonance image. Blood pool appears unusually dark on this T1 image because of gadolinium contrast uptake by amyloid deposits.

A 52-year-old male was evaluated in an emergency room because of palpitations lasting 18 hours. A 12-lead ECG revealed a regular wide-complex tachycardia with right inferior axis and right bundle branch block morphology, cycle length 400 msec and QRS duration 160 msec (Figure 1A). The tachycardia was well tolerated and was not affected by carotid sinus massage. Before institution of any other treatment, the tachycardia terminated spontaneously and was replaced by sinus rhythm with frequent ventricular ectopy and nonsustained runs of the same morphology, confirming the initial suspicion of ventricular tachycardia (VT).

The patient had no known cardiac disease or prior arrhythmia. In the preceding 3 months, he had undergone detailed cardiac evaluation for symptoms of fatigue, questionable exercise intolerance and transient ischemic attack. At that time, his transthoracic echocardiogram was reported as normal. He was able to exercise for 10 minutes on Bruce protocol. Nuclear perfusion images were indicative of anterior wall ischemia, but subsequent coronary angiogram showed normal coronary arteries. The patient was otherwise healthy except for ulcerative colitis, which required colectomy 24 years ago, and monoclonal gammopathy of unknown significance (IgG λ-light chain, 1.48 g/dL) diagnosed 14 years earlier.

Idiopathic focal VT with focus in basal anterolateral left ventricle (LV) was suspected in the emergency room, given the negative result of his recent thorough cardiac evaluation. Therefore, radiofrequency ablation was scheduled on the same day. Quadripolar electrophysiology catheters were placed in high right atrium, along the His bundle, and to right ventricular apex initially. A steerable decapolar catheter was placed into coronary sinus. A 4 mm Navistar catheter by Cordis Webster was used in conjunction with the CARTO 3D mapping system for mapping and ablation. Prior to delivery of radiofrequency energy, this patient had frequent spontaneous ventricular ectopy and nonsustained runs of VT of the same morphology as the clinical VT. No VT induction was needed. Baseline AH and HV intervals were normal. VA block was seen during 500 ms pacing.

The VT was mapped primarily with activation mapping technique. Initial mapping of right ventricular outflow tract revealed only late signals as expected. LV was accessed with the retrograde approach; LV mapping localized the focus of the tachycardia to anterobasal LV, close to mitral valve annulus. A limited CARTO map suggested a single focus. Local activity preceded onset of surface QRS by 33 ms at this site. Pacemap from this site revealed 12/12 match with clinical VT morphology. Delivery of RF energy at this resulted in marked decrease in ectopic activity, although single premature ventricular beats and occasional couplets were still observed. The residual ectopy could not be eliminated during the procedure. Only relatively low powers (10-20 W) could be delivered at this site. The patient was discharged 3 days later on oral metoprolol, which resulted in nearly complete resolution of ventricular ectopy.

**Figure 2:**
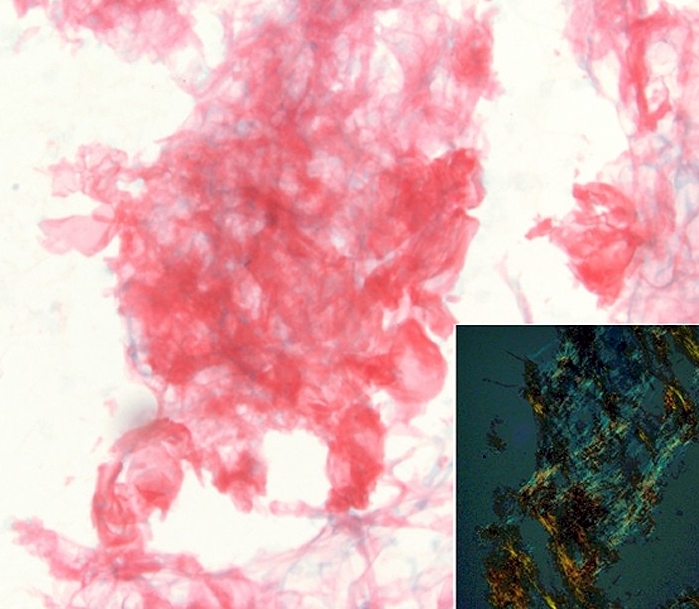
Abdominal fat pad fine needle aspiration smears stained positive with Congo red and exhibited apple-green birefringence (inset) under polarized light.

Five days after discharge, the patient was evaluated for nocturnal dyspnea and atypical chest discomfort. Physical exam revealed mild bilateral lower extremity edema, and bilateral small pleural effusions were noted on chest X-ray.  Asymmetric LV thickening, most pronounced in the posterolateral wall of the LV, was found on transthoracic echocardiogram (Figure 1B). Cardiac magnetic resonance with gadolinium contrast showed asymmetric LV thickening (15 mm posterolateral wall thickness). There was diffuse subendocardial and focal transmural enhancement on delayed images, along with unusually dark blood pool, indicative of increased gadolinium contrast clearance (Figure 1C) - a finding characteristic of amyloid cardiomyopathy [1]. Fine-needle aspirate of abdominal fat was stained with Congo red (Figure 2) and the result confirmed the diagnosis of systemic amyloidosis. The patient was started on low-dose furosemide and implanted with a dual-chamber defibrillator. There was no obvious clinical involvement of any other organ, except the bone marrow, and he underwent successful hematopoietic stem cell transplantation 6 months after presentation. The monoclonal protein is no longer detectable in the serum. His functional status is excellent (NYHA I) 18 months after presentation, with no defibrillator discharges.  His LV ejection fraction remains normal, although the asymmetric LV thickening is still detected with echocardiography.

## Discussion

Cardiac involvement is common in patients with amyloidosis due to deposition of immunoglobulin light chains [2]. Even though the heart is affected pathologically in 90% of cases, only 3.9% of cases have isolated cardiac involvement [3]. The most common presentation of cardiac amyloidosis is heart failure. The prognosis is usually poor, with median survival of 9 months after the onset of heart failure.

Approximately 30% of patients with cardiac involvement die suddenly [3]. Although many sudden deaths are caused by pulseless electrical activity, cardiac arrest due to ventricular arrhythmias is responsible in some cases [4-6]. A wide spectrum of ventricular arrhythmias occurs in patients with amyloid cardiomyopathy [7]. Nonsustained VT  is common  and correlates with poor survival [8,9]. Sustained ventricular arrhythmias have been recorded during resuscitated cardiac arrest [4] and by implantable defibrillators [5]. Radiofrequency ablation of ventricular fibrillation associated with cardiac amyloid deposition has also been reported [10]. However, well tolerated sustained monomorphic VT mimicking idiopathic VT during EP study is very unusual and has not been associated with cardiac amyloid to the best of our knowledge.  Although formal entrainment was not performed due to brief duration of VT runs during the procedure, VT in this patient behaved as a triggered rather than reentrant arrhythmia: this VT started and terminated spontaneously, without need for VT pacing induction, and focal activation sequence was suggested by activation mapping. There were no low-voltage areas suggestive of scar in the anterobasal LV region. Partial success of radiofrequency ablation was obtained at the site of perfect pacemap. All of these features would be consistent a focal, presumably idiopathic VT; the presence of cardiac amyloid provided an alternative and unusual explanation. The tachycardia was likely caused by a focus of triggered activity in the border zone of transmural amyloid infiltration.

Defibrillator implantation in amyloid cardiomyopathy is controversial because of limited survival in the affected patients [5,6]. However, the patient reported here had excellent functional status and no clinically apparent noncardiac involvement. He was felt to be a good candidate for aggressive therapy with hematopoietic stem cell transplant and potentially subsequent heart transplant [11,12]. Defibrillator implantation was thus warranted [13].
